# Subtype-specific dependencies and therapeutic opportunities in small cell lung cancer

**DOI:** 10.1126/sciadv.aea6225

**Published:** 2026-03-13

**Authors:** Amanda Luvisotto, Rima Tulaiha, Lu Wang

**Affiliations:** ^1^ Department of Biochemistry and Molecular Genetics, Feinberg School of Medicine, Northwestern University, Chicago, IL 60611, USA.; ^2^ Simpson Querrey Center for Epigenetics, Feinberg School of Medicine, Northwestern University, Chicago, IL 60611, USA.

## Abstract

Small cell lung cancer (SCLC), accounting for ~15% of lung cancers, is an aggressive and lethal tumor type. It is characterized by rapid proliferation, early metastasis, and poor prognosis. Current therapies, including platinum-based chemotherapy and recently introduced immune checkpoint inhibitors, provide modest survival benefits due to frequent relapse and therapeutic resistance. At the molecular level, SCLC is marked by near-universal loss of the tumor suppressors genes *TP53* and *RB1*, and exhibits marked heterogeneity driven by several key master transcription factors. These factors define distinct molecular subtypes with unique gene expression programs and therapeutic vulnerabilities, enabling cell plasticity and subtype switching in response to treatment pressures. A thorough understanding of these subtype-specific dependencies and the epigenetic mechanisms regulating transcription is critical for developing effective and durable therapies. This review focuses on these aspects and evaluates the potential of epigenetic-targeted strategies in the treatment of SCLC.

## Teaser

Subtype-specific dependencies and epigenetic vulnerabilities define therapeutic opportunities in small cell lung cancer.

## INTRODUCTION

Lung cancer is classified into two major categories: non–small cell lung cancer (NSCLC) and small cell lung cancer (SCLC) (*1*). SCLC is a high-grade neuroendocrine (NE) carcinoma distinguished by its aggressiveness and challenging prognosis (*2*). Although SCLC represents only about 15% of lung cancer cases, it is one of the most lethal forms of cancer, characterized by enhanced uncontrolled growth, metastatic predilection, and limited five-year survival rate (*3*, *4*). SCLC is strongly linked to tobacco exposure and shows a close association with heavy smoking history (*5*–*7*). Clinically, SCLC is classified into limited stage (LS), when the tumor is confined to one side of the chest, and extensive stage (ES), when the tumor is found beyond its original site, which makes treatment even more challenging (*8*). Notably, ~70% of patients are diagnosed at the ES, contributing to the difficulty of treatment (*9*). The pathogenesis of SCLC has traditionally been attributed to NE cells in the lung epithelium; however, emerging evidence suggests that additional epithelial cell types may also serve as potential cells of origin (*10*–*13*).

Initial molecular profiling studies of SCLC revealed that nearly all SCLC tumors harbor inactivating mutations in the tumor suppressor genes *TP53* and *RB1* (*14*–*21*). Unlike many other cancers, SCLC typically lacks frequent activating mutations and instead relies heavily on the overexpression and activity of several lineage-specific transcription factors such as ASCL1, NEUROD1, POU2F3, and YAP1 (*14*, *17*). Through the regulation of unique gene expression programs, cellular phenotypes, and therapeutic vulnerabilities, these transcriptional factors give rise to the diverse molecular subtypes that reflect the remarkable cellular and molecular SCLC heterogeneity. Moreover, the cellular plasticity driven by either transcription factors or epigenetic factors enables tumor cells to transition between molecular subtypes during progression. Although SCLCs are initially highly chemosensitive, such diversity and adaptability are thought to contribute to the development of chemoresistance and disease relapse (*22*–*25*). Therefore, understanding and targeting the transcriptional networks and epigenetic landscapes that drive SCLC heterogeneity is critical for the development of more effective and durable.

The treatment of SCLC is a challenging task, particularly due to its rapid cell growth, plastic phenotype and tendency to develop resistance to therapy (*26*). For decades, the standard treatment for extensive-stage small cell lung cancer (ES-SCLC) consisted of platinum-based chemotherapy and etoposide (*4*, *27*–*29*). More recently, immune checkpoint inhibitors, specifically PD-L1 inhibitors like atezolizumab or durvalumab, have been added to the intervention program with progress in the overall survival as demonstrated by global, phase 3 clinical trials (*29*–*33*). However, the disease often has an initial response to treatment but relapses within months and quickly becomes more resistant to later therapies (*34*–*38*). Hence, there is a pressing need for improved targeted approaches in order to improve treatment responses, avoid chemoresistance and enhance patient prognosis in SCLC.

In this review, we will discuss subtype-specific dependencies in SCLC and provide a perspective on the key molecules and signaling pathways that sustain SCLC cell identity and viability. We will also highlight the role of epigenetic factors and machinery in transcriptional regulation in SCLC cells, and the necessity of developing epigenetic therapies.

## RESULTS

### Origin and molecular classification of human SCLC

NE cells are a subset of epithelial cells present in the lungs and in many other internal organs with dual neuronal and endocrine system characteristics (*39*, *40*). These cells play fundamental roles in the maintenance of homeostasis with sensory and secretory functions (*41*). Early models proposed that NE cells are the predominant cells that give rise to SCLC, although other lung cellular types were also demonstrated to be able to originate SCLC at a reduced rate (*10*, *11*). In fact, the expression of NE markers such as synaptophysin (SYP), chromogranin A (CHGA), and calcitonin gene–related peptide (CGRP) in a large portion of SCLC tumors was identified (*42*). In particular, a rare subset of pulmonary NE stem cells activated in response to lung injury has been proposed as the cell of origin for SCLC (*43*). Using in vivo models, Ouadah and colleagues demonstrated that this NE subpopulation undergoes transformation regulated by Notch, Rb, and p53 reprogramming (*43*). Although many SCLC tumors exhibit NE dependencies, a subset shows reduced or absent expression of NE markers. For instance, tuft cells (chemosensory epithelial cells found in the lining of the airways and intestines) have been proposed as potential cells of origin for a distinct subset of SCLC displaying tuft cell–like characteristics (*13*). In a very recent study, using multiple genetically engineered mouse models (GEMMS) of SCLC, Ireland and colleagues demonstrated that a basal cell of origin can give rise to NE–tuft-like tumors, suggesting that this cell population may be a key driver of SCLC plasticity (*12*).

**Fig. 1.**
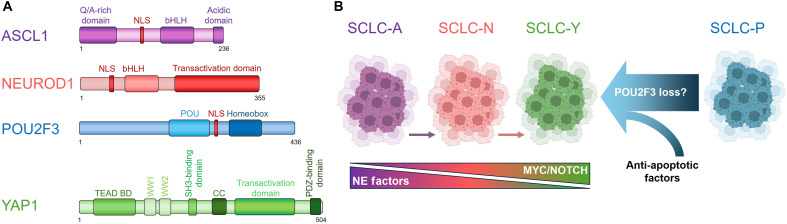
The master transcriptional regulators that define molecular subtypes of SCLC. (**A**) The schematic illustrates the domain organization of human ASCL1, NEUROD1, POU2F3, and YAP1. (**B**) The subtype plasticity of SCLC cells and subtype transition between NE SCLC cells (SCLC-A/N) and non-NE SCLC cells (SCLC-Y) mediated by MYC/NOTCH. It remains unclear how non-NE SCLC cells such as SCLC-P can convert back to NE stage.

Initially, the SCLC tumors were primarily divided into NE-high and NE-low subtypes according to the gene expression profile of NE cell markers (*44*, *45*). In modern oncology, the molecular profiling of cancers is a crucial aspect as it substantiates diagnoses, reveals molecular vulnerabilities, and guides the treatment of those malignancies (*46*, *47*). Recent studies profiling primary human and mouse tumors have further stratified NE-high and NE-low tumors into four molecular subtypes defined by the expression of key transcription regulators: ASCL1, NEUROD1, POU2F3, and YAP1 (*17*). Notably, these transcription factors share structural features, including a nuclear localization sequence, a chromatin-binding domain, and a transactivation region (Fig. 1A). The transcriptomic clustering of human primary tumor data revealed the distinct gene expression signatures of the four subtypes (*17*) (Fig. 1B). NE-high tumors, enriched for NE lineage markers, comprise ASCL1-high SCLC (SCLC-A) subtype and NEUROD1-high SCLC (SCLC-N) subtype, whereas NE-low tumors predominantly express POU2F3 (SCLC-P) or YAP1 (SCLC-Y). Notably, more recent evidence indicates that the role of YAP1 as a major transcription driver in SCLC remains uncertain. Although YAP1 expression is enriched in ASCL1/NEUROD1-negative tumors, it is broadly detected across all subtypes (*25*). Therefore, whether YAP1 can truly serve as a subtype-defining marker in SCLC warrants further clarification in future studies.

In addition to the initial subtype classification, emerging studies have suggested the segmentation of the SCLC-A, the major subtype of SCLC, into two subgroups SCLC-A1 (NE) and SCLC-A2 (NEv2). Wooten *et al.* used a computational approach to analyze transcriptomics data from SCLC cell lines and characterized four SCLC subtypes, including one NE-high variant that was previously not reported (*48*). Although this putative subtype has a high expression of ASCL1, the phenotype differs in the expression of HES1, as well as having ELF3 and NR0B1 as master transcriptional regulators, suggesting a distinct subtype SCLC-A2 (*3*, *48*). In addition, the modeling approach used in the work predicted the SCLC-A2 subtype as more chemoresistant than the other subtypes (*48*).

The evolution of profiling approaches drives an ongoing refinement of the molecular classification of SCLC (*47*). Despite the claims of inconsistent expression of YAP1 and the inability of the transcriptional factor to singly characterize a distinct SCLC subtype (*25*), recent reports using tumor expression data provide evidence of a fourth SCLC subtype mainly characterized by an inflammatory phenotype (*22*). The SCLC-I subtype, referred to as an inflammatory subtype, is primarily defined by an inflamed gene signature as well as a low expression of the other three transcription markers. Some other characteristics were also found in SCLC-I tumors, such as increased mesenchymal differentiation, decreased platinum-treatment sensitivity, higher infiltration of immune populations, and elevated expression of immune checkpoint molecules (*22*). Therefore, it is suggested that SCLC-I may benefit from immune checkpoint blockade therapy. However, due to the lack of GEMMS depicting SCLC-I, it has not been possible to determine this in vivo yet (*22*). Moreover, Nabet *et al*. analyzed RNA sequencing data from the IMpower133 trial and, using de novo non-negative matrix factorization, classified patients into distinct molecular subtypes (*49*). Notably, the study identified four subsets similar to previous characterizations, but revealed further diversity within the immune subsets, including two distinct immune-hot subsets that differ based on whether they display neuroendocrine (SCLC-I-NE) or non-neuroendocrine (SCLC-I-nonNE) features (*49*). The latter exhibited elevated expression of non-NE factors such as POU2F3 but was not composed solely of POU2F3-driven tumors and also showed reduced benefit from immune checkpoint blockade therapy in comparison with the other subtypes identified (*49*).

In addition, recent findings by Simpson and colleagues have reported an additional SCLC subtype (SCLC-AT) based on the expression of the transcription factor ATOH1, which is an NE transcription factor for SCLC tumors that regulates neurogenesis, maintains tumor cell survival, and promotes metastasis (*50*). ATOH1 is proposed as an SCLC subtype determinant (SCLC-AT) as it is expressed in a transcriptionally distinct subset of circulating tumor cell–derived explant. It is found to be either significantly expressed in SCLC samples alone or coexpressed with either ASCL1 or NEUROD1, and it exerts its function by binding to E-box motifs at promoter and enhancer regions of target genes. It was also observed that ATOH1 directly up-regulates the notch ligands and neuronal fate determination, and the reduction of ATOH1 increases the expression of non-NE genes, thus contributing to NE switch in SCLC tumors (*50*).

### Heterogeneity and plasticity of human SCLC

Initially, it was thought that SCLC represented homogenous tumors with static phenotypes (*51*). Although the expression profile of tumors classifies SCLC tumors into defined subtypes, it is believed that a temporal progression exists among these groups. Multiple studies suggest a chronological evolution between subtypes, with SCLC-A preceding SCLC-N and SCLC-Y (*24*, *52*, *53*). In this context, studies in mouse models suggest that the increased prevalence of ASCL1-high tumors reflects an in vivo prerequisite for ASCL1 to originate SCLC tumor formation (*54*). Moreover, it was found that MYC amplification is an important driver of this dynamic heterogeneity by promoting an SCLC-A to SCLC-N subtype switch, which further accelerates the tumor progression in Rb1/Trp53 null SCLC mice (*52*). In addition, Ireland *et al.* showed that MYC is implicated in this molecular SCLC subtype shift by activating the NOTCH signaling pathway and stimulating programs that direct the transformation of SCLC NE fate from an NE-high state to a non-NE state (*24*). That way, MYC is required for NOTCH activity to drive SCLC-N and SCLC-Y subtypes from SCLC-A (Fig. 1B), while SCLC-P would arise from a different cellular precursor in mice (*24*). These results indicate that the SCLC subtypes are not distinct from each other but rather are evolving stages of MYC-driven tumors (*24*). Recent studies from our group and others have shown that depletion of POU2F3 induces substantial up-regulation of either NEUROD1 or YAP1 in several SCLC-P subtype cell lines, accompanied by pronounced p53-independent programmed cell death (e.g., apoptosis) (*55*, *56*). Although the underlying molecular mechanisms remain unclear, future studies could investigate whether inhibition of cell-death signaling might enable the transition of SCLC-P subtype cells to other subtypes following the loss of POU2F3 (Fig. 1B).

Investigating this dynamic reprogramming and plasticity of tumors can also determine the therapeutic sensitivities of SCLC. Although the molecular landscape of each SCLC subtype is associated with different susceptibilities, it is thought that subtype switching may also arise from therapy resistance. For instance, distinct studies from different groups suggested that NE-low SCLC phenotypes and MYC expression were enriched after chemotherapy treatment and correlated with tumor resistance (*24*, *57*–*59*). In addition, the molecular mechanism underlying the transformation from NSCLC to SCLC (transformed SCLC or T-SCLC) during treatment also reflects SCLC subtype plasticity and could pose clinical challenges in patients (*60*, *61*). This specific transformation is usually seen to occur in epidermal growth factor receptor (EGFR)–mutant NSCLC tumors after they are treated with tyrosine kinase inhibitors or in anaplastic lymphoma kinase (ALK)–positive lung cancer cells after treatment with ALK inhibitors (*62*, *63*). Moreover, recent studies further demonstrated that TP53 and RB1 mutations can also enhance SCLC transformation in EGFR-mutant lung adenocarcinomas (*60*, *61*, *64*, *65*). Together, these data suggest that SCLC plasticity and chemoresistance are events endowed in tumor cells that pose a great challenge to SCLC treatment. Hence, the continuous investigation and profiling of SCLC molecular and biochemical dependencies is emerging as a substantial tool for directing further SCLC therapeutics.

### Identification of SCLC subtype-specific essential factors

**Fig. 2.**
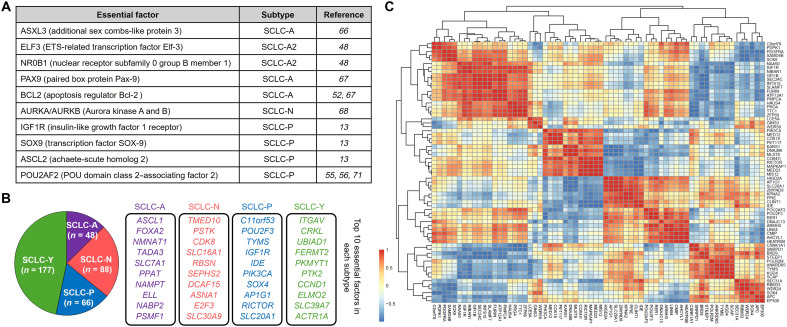
SCLC subtype–specific essential factors. (**A**) The table summarizes previous studies on essential individual factors in different SCLC subtypes. (**B**) Global identification of SCLC subtype–specific essential factors based on genome-wide CRISPR screening analysis (DepMap database), highlighting the top 10 essential factors in each subtype. (**C**) Codependency data from the DepMap database for SCLC-P subtype cells.

The subtype classification of SCLC based on the expression of major transcription factors is a well-established concept in the field; albeit the discussion regarding a fourth YAP1-positive subtype, the ASCL1, NEUROD1, POU2F3, and YAP1 (A/N/P/Y) classification remains the most widely accepted framework. Each subtype shows a unique transcriptional profile that can influence treatment response and tumor behavior (*17*). Therefore, understanding the molecular vulnerabilities of each SCLC subtype is crucial for determining personalized therapeutic approaches, especially for complex malignancies such as SCLC (*47*). As shown in Fig. 2A, a few SCLC subtype essential factors have been identified individually in the past years.

For instance, Wooten *et al*. has shown that a proposed alternative classification of SCLC, the SCLC-A2 (NEv2) subtype, was marked by the identification of ELF3 and NR0B1 as master regulators (*48*). Using network-based simulations, the research characterized master regulators of distinct SCLC subtypes as factors in which silencing led to a destabilization in subtype-specific regulatory networks, which suggests a fundamental role of ELF3 and NR0B1 in sustaining the SCLC-A2 transcriptional state (*48*).

Previous studies from our laboratory have uncovered cell type–specific epigenetic factors as essential factors for different SCLC subtypes. The ASXL transcriptional regulator 3 (ASXL3), which is specifically expressed in SCLC-A subtype cells, is critical for SCLC-A subtype cell viability by governing enhancer activity and maintaining the expression of lineage-specific genes in multiple SCLC-A subtype cell lines (*66*). Mechanistically, as a major scaffold subunit within the BAP1 complex, the ASXL3 protein bridges the histone H2AK119Ub deubiquitinase BAP1 and the bromodomain-containing protein BRD4 at active enhancers and governs enhancer activity and SCLC-A subtype-specific transcription program (*66*). Using a genome-scale CRISPR dropout screen, Zhao *et al*. identified a handful of transcription factors, such as PAX9 and B cell lymphoma 2 (BCL2), which are direct transcriptional targets of ASXL3 and critical for SCLC-A subtype cell viability (*67*). The up-regulation of the anti-apoptotic regulator B cell chronic lymphocytic leukemia/BCL2 has been observed in SCLC-A tumors, which were found to be sensitive to BCL2 inhibitor treatment or RNA interference depletion (*68*). Notably, the same study also identified that MYCL, which is frequently up-regulated or amplified in SCLC-A in contrast with other subtypes, is one of the top essential factors for SCLC-A cell types (*17*, *22*, *28*, *54*), whereas MYC single guide RNAs are significantly depleted in the surviving SCLC-N subtype cells treated with a genome-wide CRISPR library (*54*, *67*).

Similar genome-wide CRISPR screening studies in SCLC-P subtype cells have found the insulin-like growth factor 1 receptor (IGF1R) as one of the top essential factors and functioning as an activator of phosphatidylinositol 3-kinase/Akt/mammalian target of rapamycin pathway in SCLC-P cells (*13*). In addition, the same study identified the lineage transcription factors SOX9 and ASCL2 as selective vulnerabilities in POU2F3-expressing SCLC lines, revealing a cell type–specific transcription network that drives the survival of tuft cell–like SCLC tumors (*13*).

In summary, previous studies have conventionally focused on identifying essential factors on an individual basis, which presumably limits the extent of insight to isolated instances. Although this approach provides a valuable understanding, it lacks the holistic view necessary to completely comprehend the intricacy of subtype-specific essential factors throughout the genome. To overcome this limitation and globally identify and characterize all subtype-specific essential factors, we applied a systematic approach using the DepMap database (*56*). With this strategy, we uncovered a total of 379 selective essential genes across all SCLC subtypes (Fig. 2B), providing a wider and more elaborate representation of the genetic landscape of SCLC tumors (*56*). Notably, our study highlighted key essential genes as well as codependency networks (Fig. 2C) to each variant, which were enriched in distinct pathways for each subtype, indicating that different subtypes rely on unique signaling pathways for survival and proliferation (*56*).

While DepMap and other CRISPR-based screening approaches provide a powerful framework to identify essential genes and codependencies across a wide range of cancer types, as well as among distinct subtypes within a single cancer type, they also have notable limitations. Data generated from whole-genome CRISPR screens can be influenced by variable guide efficiency, off-target effects, and cell line–specific variability (*69*). Furthermore, the reliance on established cell lines limits the representation of tumor heterogeneity, meaning that in vitro studies may not fully capture the complexity of in vivo tumor biology or reflect the full diversity of patient tumors (*69*). Therefore, in vivo, spheroid-, or organoid-based whole-genome screening platforms should be developed and systematically characterized to better account for physiological conditions and tumor microenvironmental factors (*69*, *70*). Nevertheless, these studies highlight critical vulnerabilities that, upon further validation, may offer promising therapeutic opportunities for SCLC.

### POU2AF2 (OCA-T1): A previously unidentified coactivator of POU2F3 and top essential factor for the SCLC-P subtype

In light of the global characterization of subtype-specific essential factors, our group as well as others revealed a previously uncharacterized gene in SCLC-P subtype cells, initially named *C11orf53*, which has been identified as the top essential factor for this particular subtype (*55*, *56*, *71*). Depletion of this gene by CRISPR leads to a dramatic alteration of transcription in SCLC-P cells, resulting in a profoundly catastrophic cell death in variant SCLC-P subtype tumors (*56*), which comprises about 16% of SCLC tumors (*17*) and has a relatively poorer prognosis compared to the other three subtypes (*22*).

**Fig. 3.**
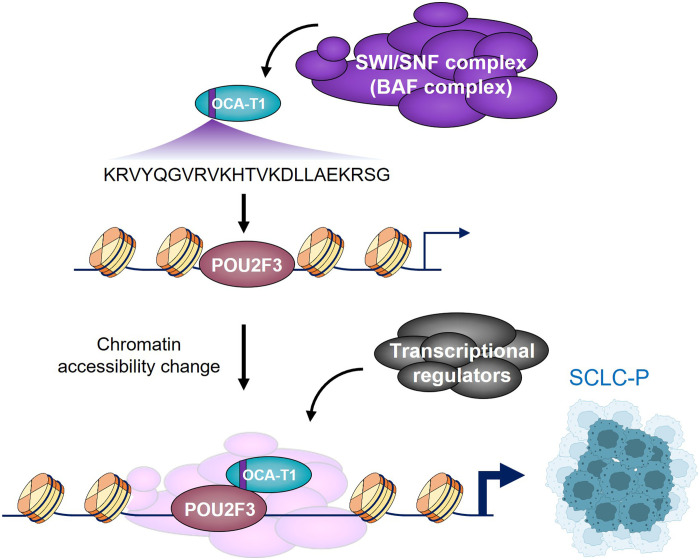
The function of POU2F3/POU2AF2 heterodimer in SCLC-P subtype. The POU2AF2 protein does not directly bind to chromatin due to the absence of a chromatin-binding domain. Instead, it may be recruited to chromatin via an N-terminal OCA peptide that directly interacts with POU2F3. The C-terminal region of POU2AF2 is highly disordered and functions as a transactivation domain, potentially recruiting or facilitating the recruitment of epigenetic factors such as the SWI/SNF complex to specific chromatin regions.

Based on the codependency analysis, *C11orf53* is the top codependent gene with *POU2F3* in SCLC-P subtype cells (Fig. 2C). Biochemical studies from our laboratory and our colleagues further demonstrated that the protein encoded by *C11orf53* directly interacts with POU2F3 in vitro and in SCLC cell lines through its N terminus, which contains a conserved motif shared with a known coactivator, POU2AF1 (also known as BOB1/OCA-B) (Fig. 3) (*55*, *56*, *72*). The in vitro gel filtration assay has shown that *C11orf53* encoded protein is able to facilitate the formation of POU2F3/DNA complex, although POU2F3 is able to bind to DNA independently. At genome-wide levels, the protein encoded by *C11orf53* occupies a typical POU family-bound octamer motif ATGCAAAT, and functions as a robust transcriptional coactivator of POU2F3 at distal enhancer elements and maintains the expression levels of the vast majority of POU2F3 target genes (*55*, *56*). Therefore, the *C11orf53* gene was subsequently renamed *POU2AF2* (*56*), followed by the name of the first coactivator of the POU2 family transcription factor, POU2AF1 (*73*), to reflect its association and functional partnership with POU2F3. The protein product of *C11orf53* gene was further named as OCA-T1 due to its essential function in both tuft cells and tuft cell–like tumors such as SCLC-P (*55*).

At the chromatin levels, the OCA-T1/POU2F3 heterodimer is able to recruit other epigenetic machinery to maintain the expression of SCLC-P specific transcriptional program. For instance, depletion of either OCA-T1 or POU2F3 leads to a dramatic redistribution of the SWI/SNF chromatin remodeling complex (also known as the BAF complex) in multiple SCLC-P cells, leading to a substantial change of three-dimensional genome architecture and gene expression (Fig. 3) (*56*). Additional acute protein degradation technologies should be considered to further understand how the rapid degradation of POU2AF2 affects the chromatin recruitment of other epigenetic and transcription factors in SCLC-P cells. Last, it appears that OCA-T1 is able to transcriptionally activate POU2F3 expression at the chromatin and vice versa, suggesting a potential positive feedback mechanism between the transcription factor and the coactivator in SCLC cells. Future studies may focus on whether OCA-T1 is sufficient to transform other cell types into tuft cells through its interaction with POU2F3.

### Global therapeutic strategies for targeting SCLC

Currently, the treatment of SCLC depends largely on the stage of the disease and overall involves a combination of radiotherapy, chemotherapy agents, and immunotherapy (*27*, *29*). Limited-stage small cell lung cancer (LS-SCLC), in which the tumor is restricted to one side of the thorax, is managed with concurrent radiotherapy and chemotherapy (a platinum-based agent alongside a topoisomerase inhibitor, etoposide) (Table 1) (*27*, *29*, *74*). Recent clinical results from Cheng *et al*. led to the incorporation of durvalumab (a PD-L1 inhibitor) following chemoradiation as an updated standard of care for LS-SCLC, given the remarkably longer overall survival and progression-free survival of LS-SCLC patients treated with the adjuvant therapy (*75*). For ES-SCLC, in which the tumor has spread outside this area and often includes distant metastases, the treatment consists of longer cycles of combined chemotherapy (carboplatin or cisplatin with etoposide) and immunotherapy (particularly PD-L1 blockers such as atezolizumab and durvalumab) (*8*, *27*, *29*, *30*, *33*, *74*, *76*). However, although these regimens often produce initial responsiveness, relapse is frequently observed in ES-SCLC, with often increased resistance to therapy (*33*–*38*, *77*–*80*). Therefore, identifying molecular dependencies in cancer will pave the way for finding additional treatable targets, with important implications for improved therapeutic strategies.

**Table 1.** Representative current and in-development treatment for SCLC.

Name	Target	Type	Stage/predicted benefit	Reference
**Current treatment**				
Etoposide	TOPO II	Chemotherapy	ES-SCLC standard of care	*27*–*29*
Cisplatin	DNA	Chemotherapy	ES-SCLC standard of care	*27*–*29*, *74*
Carboplatin	DNA	Chemotherapy	ES-SCLC standard of care	*27*–*29*, *74*
Durvalumab	PD-L1	Immunotherapy	LS-SCLC standard of care	*75*
Atezolizumab	PD-L1	Immunotherapy	ES-SCLC first-line treatment	*30*
Tartalamab	DLL3 and CD3	Immunotherapy	Relapsed ES-SCLC	*93*, *94*
**Treatment in development**				
CAR T	DLL3	Immunotherapy	SCLC-A	*90*, *91*
ORY-1001	LSD1	Target therapy	ES-SCLC	*87*–*89*
GSK126	EZH2	Target therapy	ES-SCLC	*86*
Venetoclax	BCL2	Target therapy	SCLC-A	*96*, *97*
Rovalpituzumab tesirine	DLL3	Target therapy	SCLC-A	*92*
AURKi (alisertib/barasertib)	Aurora kinase	Target therapy	SCLC-N	*52*
PARPi (olaparib/veliparib)	PARP1/2	Target therapy	SCLC-P	*22*, *99*
Dalotuzumab/cixutumumab	IGF1R	Target therapy	SCLC-P	*100*, *101*

Despite the incorporation of immunotherapy to the SCLC standard of care, the disease continues to exhibit deficient major histocompatibility complex I (MHC-I) antigen presentation, a key mechanism of immune escape that is proposed to result from epigenetic programming (*81*–*85*). Consequently, epigenetic strategies aimed at restoring MHC-I expression and enhancing tumor immunogenicity have emerged as potential therapeutic approaches. For example, Mahadevan and colleagues demonstrated that inhibition of EZH2 can epigenetically restore MHC-I expression, thereby enhancing tumor immunogenicity in vivo (*86*). Similarly, the lysine-specific histone demethylase 1 (LSD1), which interacts with transcriptional repressors including histone deacetylase 1/2 (HDAC1/2) and CoREST, has been implicated in suppressing antigen presentation (*87*). In a recent study, Nguyen *et al*. reported that LSD1 inhibition reactivates MHC-I expression, promotes antigen presentation and interferon signaling, and consequently enhances tumor immunogenicity. Notably, LSD1 inhibition also induces the loss of NE features in NE SCLC subsets, and its combination with immune checkpoint blockade further augments antitumor immune responses (*88*, *89*).

In addition, studies have identified a transmembrane protein named delta-like ligand 3 (DLL3) as a promising immunotherapy target due to its selective cell-surface expression in SCLC and strong preclinical evidence supporting the efficacy of DLL3-directed chimeric antigen receptor (CAR) T cells and bispecific T cell engager (BiTE) antibodies (*90*–*93*). Tarlatamab, a first-in-class BiTE targeting DLL3 and CD3, has demonstrated notable antitumor activity and a favorable safety profile in patients with relapsed or refractory SCLC, leading to its accelerated US Food and Drug Administration approval for the treatment of relapsed ES-SCLC (*93*, *94*). By simultaneously binding CD3 on T cells and DLL3 on tumor cells, tarlatamab redirects cytotoxic T cells toward cancer cells, promoting the formation of cytolytic synapses and antigen-dependent tumor cell lysis while disrupting tumor-promoting signaling (*93*).

### Subtype-specific therapeutic targeting of SCLC

Despite recent advances in the treatment of SCLC, outcomes remain poor due to its aggressive behavior and remarkable heterogeneity. Increasing evidence underscores the need for personalized therapeutic strategies that account for the molecular and phenotypic diversity among SCLC subtypes. As consolidated in Table 1, distinct SCLC molecular subtypes exhibit different vulnerabilities that can yield subtype-specific therapeutic strategies. For instance, the SCLC-A subtype, which represents the largest proportion of SCLC cases (*22*, *25*, *95*), exhibits classical NE features and high expression of the anti-apoptotic protein BCL2. As a result, SCLC-A tumors have shown sensitivity to BCL2 inhibitors, such as venetoclax, which is currently being evaluated in clinical trials (*22*, *96*, *97*). However, the clinical implementations of venetoclax have been impaired due to the toxicity profile of this drug; thus, further strategies are yet to be developed (*96*, *98*).

In contrast, the SCLC-N subtype represents about 30% of SCLC tumors (*95*). MYC amplifications are frequently observed on this subtype, driving proliferation and dependency (*22*). Consequently, Aurora kinase inhibitors, in particular Aurora A and B inhibitors, have shown notable efficacy in SCLC-N and MYC-amplified samples, especially when combined with standard chemotherapy (*22*, *52*). Furthermore, additional MYC-regulated vulnerabilities, such as metabolic reprogramming, particularly arginine-regulated pathways, are also emerging as promising targets in SCLC-N (*58*).

Despite the ongoing debate over a non-NE inflamed subtype of SCLC, a fourth, rarest SCLC subtype (characterized by low ASCL1, NEUROD1, and POU2F3 expression) showed significantly higher RNA and protein expression levels of PD-L1 as compared to the other SCLC cell lines, and PD-L1 expression levels increased with overexpression of YAP1 in YAP1-positive cells, which subsequently inhibits T cell infiltration and function, leading to an immunosuppressive environment (*22*). Therefore, therapeutically cotargeting YAP1 and PD-L1 in those YAP1-positive SCLC tumors could be an effective combination immunotherapy in YAP1-positive patients and could further enhance antitumor activity.

The SCLC-P subtype accounts for ~16% of all SCLC cases and is associated with poorer prognosis and reduced response to chemotherapy (*55*, *56*, *71*). Initially, it was found that SCLC-P models were more sensitive to various types of poly(adenosine diphosphate–ribose) polymerase (PARP) inhibitors (PARPi), compared to other subtypes (*22*). However, the underlying molecular mechanisms remain to be uncovered (*99*). Therefore, multiple genome-wide CRISPR screenings have been applied to globally identify factors/signaling pathways that are essential for this subtype to facilitate the development of previously unidentified therapeutics. For instance, the receptor tyrosine kinase IGF1R has been identified as a key determinant of cell identity in SCLC-P cells based on kinase domain–focused CRISPR screening (*13*). IGF1R expression levels across SCLC cell lines do not correlate well with their dependency on IGF1R, a pattern consistent with most other subtype-specific dependencies (*56*). This observation underscores the fact that SCLC-P subtype cells are particularly reliant on IGF1R-mediated signaling. Consequently, multiple humanized monoclonal antibodies targeting IGF1R have been developed and evaluated as potential therapeutic strategies for SCLC-P subtype tumors (*100*, *101*).

Recently, studies from our group and others have uncovered the central role of OCT-T1, the previously unidentified coactivator of POU2F3, in regulating the chromatin landscape in SCLC-P tumor cells through its interaction with the SWI/SNF complex (*55*, *56*, *71*, *102*–*104*). Genome-wide CRISPR and proteomic analysis revealed both physical and functional associations between OCA-T1 and multiple SWI/SNF subunits (*102*–*104*). As a result, inhibition of the SWI/SNF complex by its specific small molecule inhibitor or degrader impairs SCLC-P cell viability in vitro and in animal models, supporting the dependency of SCLC-P on this chromatin remodeling machinery (*103*, *104*).

## DISCUSSION

Over the past few decades, the development of therapies for SCLC has seen modest progress compared to other lung cancer subtypes. SCLC has been treated with platinum-based chemotherapy, which remains the standard of care due to its initial high response rate (*2*, *30*, *105*). Efforts to improve outcomes have included the introduction of radiation therapy, prophylactic cranial irradiation, and recently developed immune checkpoint inhibitors such as atezolizumab and durvalumab, which have provided some benefits and offered a modest survival advantage in LS disease (*33*, *106*). Despite these efforts, the aggressive nature and rapid progression of SCLC continues to pose a remarkable therapeutic challenge. Given the limited efficacy of current treatment options and the rapid development of resistance in SCLC, there is a critical need to explore additional therapeutic strategies, including epigenetic-targeted therapies and improved combination therapy approaches. Recent emerging evidence suggests that epigenetic drugs, such as inhibitors of HDACs, DNA methyltransferases, and LSD1, can suppress SCLC growth, alter tumor cell identity, and improve responses to chemotherapy and immunotherapy (*24*, *107*, *108*). Therefore, further understanding the underlying function of epigenetic machinery and chromatin modifications as well as the development and integration of epigenetic therapies hold promise for overcoming therapeutic resistance and improving long-term outcomes in SCLC patients.
